# Gaseous NO_2_ induces various envelope alterations in *Pseudomonas fluorescens* MFAF76a

**DOI:** 10.1038/s41598-022-11606-w

**Published:** 2022-05-20

**Authors:** Thibault Chautrand, Ségolène Depayras, Djouhar Souak, Tatiana Kondakova, Magalie Barreau, Takfarinas Kentache, Julie Hardouin, Ali Tahrioui, Olivier Thoumire, Yoan Konto-Ghiorghi, Corinne Barbey, Guy Ladam, Sylvie Chevalier, Hermann J. Heipieper, Nicole Orange, Cécile Duclairoir-Poc

**Affiliations:** 1 Research Unit Bacterial Communication and Anti-Infectious Strategies (UR CBSA), Normandy University, Univeristy of Rouen Normandy, 55 rue Saint-Germain, 27000 Evreux, France; 2 Praxens, Normandy Health Security Center, 55 rue Saint-Germain, 27000 Evreux, France; 3 LPS-BIOSCIENCES SAS, Domaine de l’Université Paris Sud, Bâtiment 430, Université Paris Saclay, 91400 Orsay, France; 4 Polymers, Biopolymers, Surface Laboratory, Normandy University, University of Rouen Normandy, INSA Rouen, CNRS, Bâtiment DULONG – Bd Maurice de Broglie, 76821 Mont Saint Aignan Cedex, France; 5 PISSARO Proteomic Facility, IRIB, 76820 Mont-Saint-Aignan, France; 6 Polymers, Biopolymers, Surface Laboratory, Normandy University, University of Rouen Normandy, INSA Rouen, CNRS, 55 rue Saint-Germain, 27000 Evreux, France; 7 Department of Environmental Biotechnology, Helmholtz Centre for Environmental Research – UFZ, Permoserstraße 15, 04318 Leipzig, Germany

**Keywords:** Membrane structure and assembly, Bacterial physiology, Air microbiology, Membrane lipids, Phospholipids, Flow cytometry, Reverse transcription polymerase chain reaction, Proteomic analysis, Fluorescent dyes, Membranes, Scanning electron microscopy

## Abstract

Anthropogenic atmospheric pollution and immune response regularly expose bacteria to toxic nitrogen oxides such as NO^•^ and NO_2_. These reactive molecules can damage a wide variety of biomolecules such as DNA, proteins and lipids. Several components of the bacterial envelope are susceptible to be damaged by reactive nitrogen species. Furthermore, the hydrophobic core of the membranes favors the reactivity of nitrogen oxides with other molecules, making membranes an important factor in the chemistry of nitrosative stress. Since bacteria are often exposed to endogenous or exogenous nitrogen oxides, they have acquired protection mechanisms against the deleterious effects of these molecules. By exposing bacteria to gaseous NO_2_, this work aims to analyze the physiological effects of NO_2_ on the cell envelope of the airborne bacterium *Pseudomonas fluorescens* MFAF76a and its potential adaptive responses. Electron microscopy showed that exposure to NO_2_ leads to morphological alterations of the cell envelope. Furthermore, the proteomic profiling data revealed that these cell envelope alterations might be partly explained by modifications of the synthesis pathways of multiple cell envelope components, such as peptidoglycan, lipid A, and phospholipids. Together these results provide important insights into the potential adaptive responses to NO_2_ exposure in *P. fluorescens* MFAF76a needing further investigations.

## Introduction

Reactive nitrogen species (RNS) form a group of highly reactive compounds that include nitric oxide (NO^•^), nitrogen dioxide (NO_2_) and peroxynitrite (ONOO^−^). NO^•^ and NO_2_ are major pollutants resulting from fuel combustion processes used in the industry and transport. These molecules are responsible for environmental problems such as acid rain, photochemical smog and ozone depletion^1^. RNS can react with a wide range of biological molecules, such as DNA^2^, proteins^3^ and lipids^4,5^. Indeed, NO^•^ can induce nitrosative deamination of DNA bases and sugar oxidative modifications of DNA^2^. Meanwhile, peroxynitrite can act as a strong oxidant toward thiols and transition metal centers, and can induce tyrosine nitration^3^. Furthermore, NO_2_ has the ability to induce lipid peroxidation^5^. This ability to damage biomolecules makes RNS toxic for biological organisms at high concentrations. RNS concentration in the atmosphere is highly dependent on the local polluting human activities as well as the climatic conditions^6–8^. For human health, the World Health Organization fixes the annual guideline value at 0.1 ppm, and NO_2_ is considered to have reversible effects on human health after 1 h of exposition to 5 ppm, and irreversible effects after 1 h at 45 ppm^9,10^.

In the environment, microorganisms can be exposed to RNS from various sources, such as the atmosphere or the immune response, as well as collateral products of their own metabolism^11,12^. Some bacterial species possess denitrification pathways allowing them to use these species as terminal electron acceptors for their energetic metabolism^12–14^. The mechanisms developed by bacteria to counteract the effects of RNS are tightly linked to their virulence. Indeed, during the primary immune response, macrophages produce a burst of RNS and reactive oxygen species (ROS) to eliminate pathogens^11^. Thus, resistance to RNS is often a crucial part of the infectious process^15,16^.The first bacterial component to encounter exogenous RNS is the bacterial envelope. In Gram negative bacteria, this envelope is constituted by two lipid bilayers separated by the periplasmic space containing the peptidoglycan layer. The stability and functions of these membranes are the result of complex interactions between lipids and proteins. Indeed, membrane proteins functionality is often dependent on the steric hindrance caused by nearby lipids^17,18^. Membranes play an important role in the context of nitrosative stress^4^. NO^•^ and NO_2_, the most common RNS found in the environment, is more soluble in the hydrophobic core of the membranes than in water^4^. Their great concentration in the membrane enhances their reactivity with other RNS and reactive oxygen species (ROS) such as O_2_^●−^, which reacts with NO^•^ to form the extremely reactive ONOO^−^^19^. Furthermore, NO_2_ can react with fatty acids possessing at least two unsaturations in several ways such as peroxidation, nitration and nitrosylation^4^.

*Pseudomonas fluorescens* is considered a ubiquitous bacterium with a very adaptable metabolism. The *P. fluorescens* MFAF76a strain was isolated from air and possesses several virulence factors, such as the secretion of lipases and proteases, the ability to form biofilms at 37 °C and a high lytic activity on the human pulmonary cell line A549^20^. These characteristics make the strain MFAF76a a good model to study the effects of atmospheric NO_2_ pollution on bacteria. The objectives of this study were to assess the physiological impact of gaseous NO_2_ on the bacterial cell envelope and to characterize the potential molecular mechanisms underlying the adaptive responses to NO_2_ exposure in *P. fluorescens* MFAF76a.

## Materials and methods

### Bacterial strain

The bacterial strain used in this study is the Gram negative *P. fluorescens* MFAF76a, an airborne strain isolated from dust clouds of the Rouen harbor installations (Normandy, France) generated during crop ship loading^20^.

### Exposition to NO_2_

*P. fluorescens* strain MFAF76a was precultured at 28 °C for 24 h in Lysogeny Broth (LB) (yeast extract 5 g/L, peptone 10 g/L, NaCl 5 g/L). Precultures were used to inoculate strain MFAF76a at a final optical density at 580 nm (OD_580_) of 0.08 in Davis Medium Broth (DMB) supplemented with 2.16 g/L of glucose^21^. Culture was incubated at 28 °C during 16 h with agitation at 180 rpm. Bacteria were then centrifuged at 13,000 g for 10 min at 4 °C and resuspended in DMB at an OD_580 nm_ value of 4. Then, 125 µL of bacteria were deposited on nitrocellulose filters (Pore size 0.2 µm, diameter 47 mm, Sartorius Stedim Biotech GmbH, Germany) themselves deposited on Petri dishes containing DMB and 15 g/L agar. Bacteria were incubated 4 h at 28 °C and the filters were deposited on 15 g/mL agar plates, and placed into the exposition system described by Kondakova and coworkers^21^. Bacteria were exposed to 45 ppm of gaseous NO_2_ at a N_2_/O_2_ ratio of 8/2 (v/v) or synthetic air with the same N_2_/O_2_ ratio for 2 h with a constant gas stream of 2 L/min. Exposition to NO_2_ and air were limited to 2 h to prevent excessive drying of the cells, as well as for time constraints. After exposure, bacteria were resuspended in sterile physiological water (NaCl 9 g/L). The collected exposed (45 ppm NO_2_) and control (synthetic air) bacterial suspensions were then used for all subsequent experiments.

### Electronic microscopy

The sample preparation method for electronic microscopy was adapted from Bergeau et al.^22^. After exposition to NO_2_ or synthetic air, 1 mL of bacterial suspension was centrifuged 5 min at 8,000 g at room temperature (RT). Supernatant was discarded and bacteria were resuspended into 100 µL of PBS. 50 µL were deposited onto electron microscopy grids for 5 min. The suspension was discarded, and the cells were fixated for 20 min with 50 µL of fixation solution (Paraformaldehyde 2%, Glutaraldehyde 0.2%, PBS). Bacteria were then washed three times by PBS for 5 min and washed three times with deionized water. Fixated bacteria were then observed by scanning electronic microscopy (JEOL JCM-6000, Tokyo, Japan). Bacteria with apparent envelope alterations were manually counted using ImageJ software version 1.52a (5 images were taken per experiment, N = 3), and their number was divided by the total number of cells^23^. Only cells that were fully visible on the pictures were taken into account.

### Flow cytometry assay

The exposed and control bacterial suspensions after exposure were stained for 15 min with propidium iodine 30 nM (PI) and SYTO9™ 5.01 nM using the Live/Dead *Bac*Light kit (L-7012, Thermofisher, United States) according to the manufacturer instructions. Bacteria were observed using flow cytometry (CytoFLEX S, Beckman Coulter, United States) and the Cytexpert v1.2 software. PI and SYTO9™ were excited at a wavelength of 488 nm and their fluorescence emissions were detected at 690 ± 50 nm and 525 ± 40 nm, respectively. A total of 10,000 events were collected for each measurement at a low flow (10 µL/min). Positive control for membrane integrity was performed on bacteria by treating bacteria with 50% ethanol for 10 min prior to staining. The software was calibrated using the positive integrity control.

### Whole proteome analysis

The bacterial suspension after exposition was centrifuged at 13,000 g at 4 °C for 20 min. Pellets were then resuspended in 20 mM Tris–HCl buffer (pH 7.4) and sonicated. Bacterial lysate was centrifuged at 10,000 g at 4 °C for 10 min, and their protein concentration was determined by Bradford test (Biorad™, United States). Total proteins were identified as described previously^24^. Briefly, proteins were loaded onto an SDS-PAGE stacking gel (7%). After short electrophoresis migration, proteins were reduced, alkylated and digested with trypsin. After extraction, peptides were injected on a LTQ-Orbitrap Elite mass spectrometer coupled to an Easy nLC II system (Thermo Scientific, United States). The mobile phase was composed of 0.1% formic acid (FA) in H_2_O (buffer A) and 0.1% FA in acetonitrile (buffer B). Samples were injected onto an enrichment column (C18 Acclaim PepMap100, Thermo Scientific, United States) and eluted using a three-step linear gradient (from 2 to 40% B over 75 min, from 40 to 80% B in 4 min, and 11 min at 80% B) at a flow rate of 300 nL/min. The samples were analyzed using CID (collision induced dissociation) method. All measurements in the Orbitrap analyzer were performed with on-the-fly internal recalibration (lock mass) at m/z 445.12002 (polydimethylcyclosiloxane). After MS analysis, raw data were imported in Progenesis LC–MS software (Nonlinear Dynamics, Netherlands). For comparison, one sample was set as a reference and the retention times of all other samples within the experiment were aligned. After the statistical analysis was performed, peptide features presenting a p-value and a q-value less than 0.05, and a power greater than 0.8 were retained. MS/MS spectra from selected peptides were exported for peptide identification with Mascot (Matrix Science, United Kingdom) against the database restricted to *P. fluorescens* A506 (http://www.pseudomonas.com).

### Membrane fluidity assay

The exposed (NO_2_ 45 ppm) and control (synthetic air) bacterial suspensions were washed 3 times with MgSO_4_ 10 mM by centrifugation at 13,000 g for 5 min at RT and resuspension of the pellets in a solution of 10 mM of MgSO_4._ Bacteria were then resuspended in MgSO_4_ 10 mM and adjusted to an OD_580_ value of 0.1. Next, bacterial membranes were labelled using 1,6-diphenyl-1,3,5-hexatriene (DPH) fluorescent probe at a final concentration of 4 µM of DPH and incubated in darkness at 28 °C for 30 min. Fluorescence anisotropy was measured with a Spark 20 M multimode microplate reader (Tecan Group Ltd., Männedorf, Switzerland Inc., Research Triangle Park, N.C.). Steady state anisotropy was calculated using Eq. (1):

1 $$r = \frac{{I_{vv} - GI_{vh} }}{{I_{vv} + 2GI_{vh} }},$$

where *I* represents fluorescence intensity, and “*v*” and “*h*” respectively represent vertical and horizontal settings of the excitation and emission polarizers. The instrumental correction factor *G* was calculated separately for each experiment by Eq. (2):

2 $$G = \frac{{I_{hv} }}{{I_{hh} }}.$$

### Quantitative reverse-transcription real time polymerase chain reaction (RT-qPCR)

Total RNAs were isolated either from pellets of bacteria exposed (45 ppm NO_2_) or control (synthetic air) using the hot acid-phenol method^25^. Residual genomic DNA was eliminated using Turbo DNA-free kit (Invitrogen, United States). RNAs were non-specifically converted to single stranded cDNAs using the High-Capacity cDNA Reverse Transcription Kit following manufacturer’s protocol (Applied Biosystems, United States). RT-qPCR experiments were performed as previously described^26^ using the primers listed in Table 1. The relative quantification of the mRNAs was obtained by the comparative CT (2^–ΔΔCT^) method as described in^27^. Measurement of *recA* gene expression was used as an internal standard. *recA* has stable expression within the samples to be compared, regardless of physiological or experimental conditions^28^.

**Table 1:** List of primers used for qRT-qPCR experiments.

Gene	GenBank accession number	Forward primer sequence (5′ > 3′)	Reverse primer sequence (5′ > 3′)
*pgpA*	MT815580	CCGGAAGGCTGGTACTGGTT	GAGAATATCGAAGAAGCGGAACA
*pgsA*	MT815581	GCAGGTGGCCGTTTCTAATC	AATCACCAGCGCGAGCAT
*clsA*	MT815582	ACGGCACCGCGACCTT	TACGGCCTCCTTCGCTTGT
*clsB*	MT815583	TGAGCCTGTCGCTGAATCTG	AGTGGTTCTGGCTGAGGTCTTC
*cdsA*	MT815584	AAGTTCGGCAAGCGCAAA	GTAAACGCCTTCCCAGCTCTT
*pssA*	MT815585	GCGCTGCCGTATCTGTATGA	GCTGTCGATGCTGTGCTGAT
*pcs*	MT815577	GGCGATACCGGCTTGCTT	TGGCGATGTGTACGGTTGA
*psd*	MT815578	TGGTTTGCCAAGCGTTATCA	AAGGCGTTGAAGTGCTCGTAAG
*clsC*	MT815579	GTGGCTGGCGGACAACA	TGTCGATGTCGGTGAAATTCA
*plsC*	MW930719	TCTGCGCCCGCCTCTA	CCGTTGACCTCCGACTTGA
*ftsA*	MT643914	CGACGAACTGTTCACCCTGA	GGATCAGGTCTTCGTAGCCG
*ftsZ*	MT643915	TGACCATCCTCGGCAAAGAC	GCCAGTACATCGTCAGCCTT
*ftsE*	MT643916	GCTTGCATGAGCTGAGCTTT	GGCCGGTGACAAACAAGAAC
*FtsI*	MH937718	GATGTGAAGACCGGCGAGAT	TTGTTCGGGTTGTAGGTCGG
*minE*	MT643917	TGACTTCTTTCGTGCCAACAA	GCTGGCCGCGCTCAT
*mraZ*	MW930718	AATTGTGGGACGAGGATGCA	GCGCCCGGTTGTTGAA
*recA*	MH937724	ATCGCCCATACGCATTACG	CGGCCCTGGGTCAGATC

### Statistical analyses

All experiments were repeated at least three times. Mann–Whitney test was used to determine the significance between mean values of the cell sizes obtained by flow cytometry. Two tailed unpaired t-test was used to determine the significances of differences between mean values for the live/dead cytometry, fluorescence anisotropy and RT-qPCR experiments. Significance was set at p < 0.05 (*), p < 0.01(**) and p < 0.001 (***). For the proteomic study, after alignment and normalization, statistical analysis was performed using one-way analysis of variance (ANOVA) calculations. The significance of the peroxynitrite labelling was determined using two-way ANOVA calculations.

## Results

### NO_2_ exposure alters* P. fluorescens* MFAF76a cells morphology

**Figure 1:**
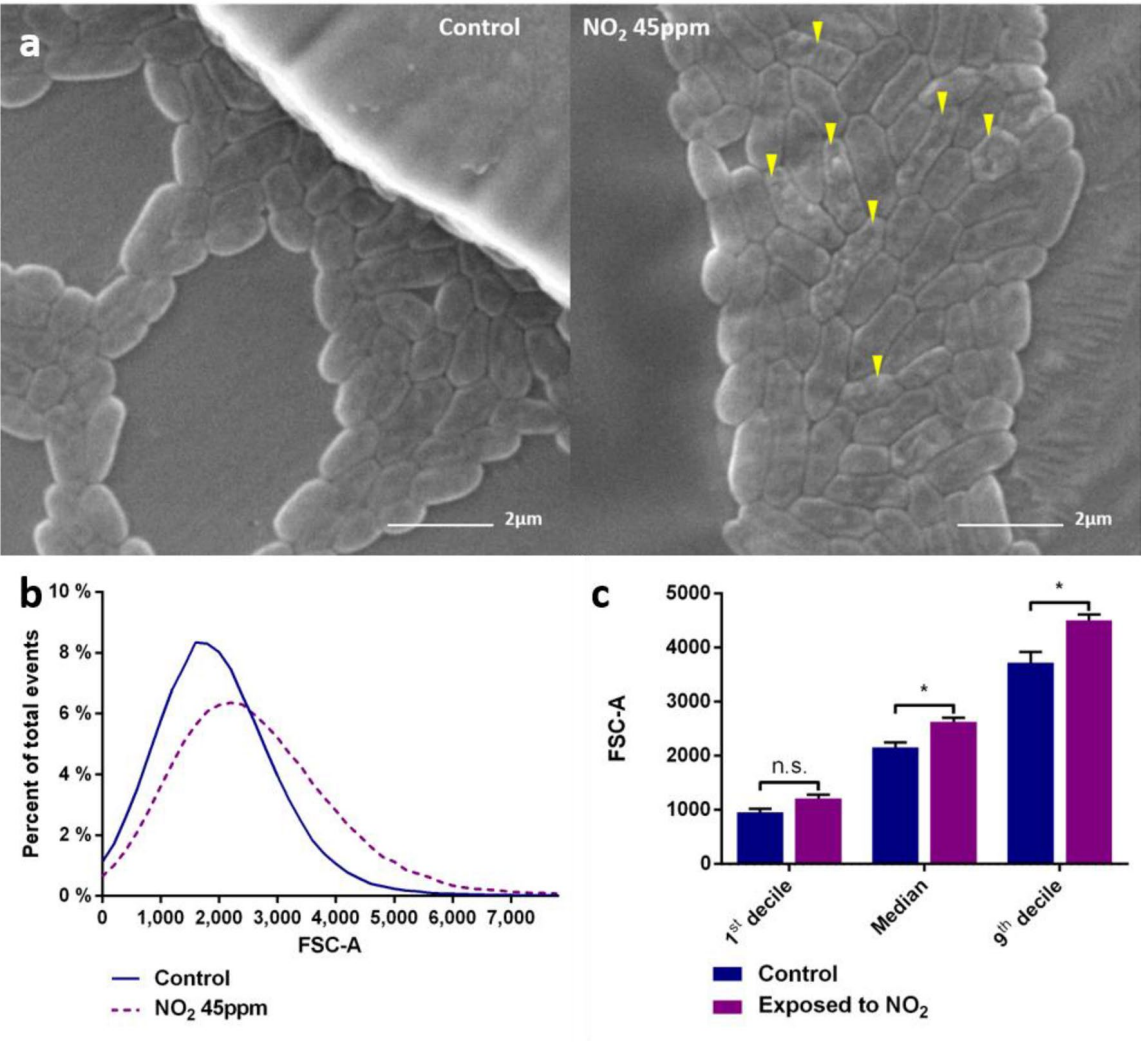
Morphology of *P.fluorescens* MFAF76a after exposure to NO_2_ 45 ppm. (**a**) *P. fluorescens* MFAF76a strain observed by scanning electron microscopy. After exposure to either synthetic air (control) or 45 ppm of NO_2_ (NO_2_ 45 ppm) for 2 h, *P. fluorescens* MFAF76a were fixed and observed by scanning electron microscopy, revealing envelope alterations (yellow arrows). (**b**) Forward scatter (FSC-A) curve of MFAF76a cells exposed to 45 ppm of NO_2_ (NO2 45 ppm) or synthetic air (Control). (**c**) Forward scatter (FSC-A) of the 1st, 5th and 9th deciles of MFAF76a cells exposed to 45 ppm of NO_2_ or synthetic air. Statistical significance was determined using a Mann–Whitney test (N = 5). n.s. = *p* > 0.05 * = *p* < 0.05.

Previous work by T. Kondakova and coworkers determined that *P. fluorescens* MFAF76a showed significant modifications when exposed to 45 ppm of NO_2_, and we therefore chose to focus on this concentration^21^. To determine if NO_2_ exposure changes the cell morphology, *P. fluorescens* strain MFAF76a cells were observed by scanning electron microscopy after exposure to 45 ppm of NO_2_ (Fig. 1a). Around 35% of bacteria exposed to NO_2_ displayed impaired cell surface as compared to the control (Fig. 1a). The aspect of altered cell envelopes was rough and bumpy, presenting asperities and potential cavities. Flow cytometry assay analyses showed that *P. fluorescens* MFAF76a cells exposed to NO_2_ had a significant increase of the forward scatter (FSC), which indicates an augmentation of their size (Fig. 1b,c).

### NO_2_ exposure modifies the quantities of proteins involved in divisome formation as well as in the synthesis of envelope components

**Figure 2:**
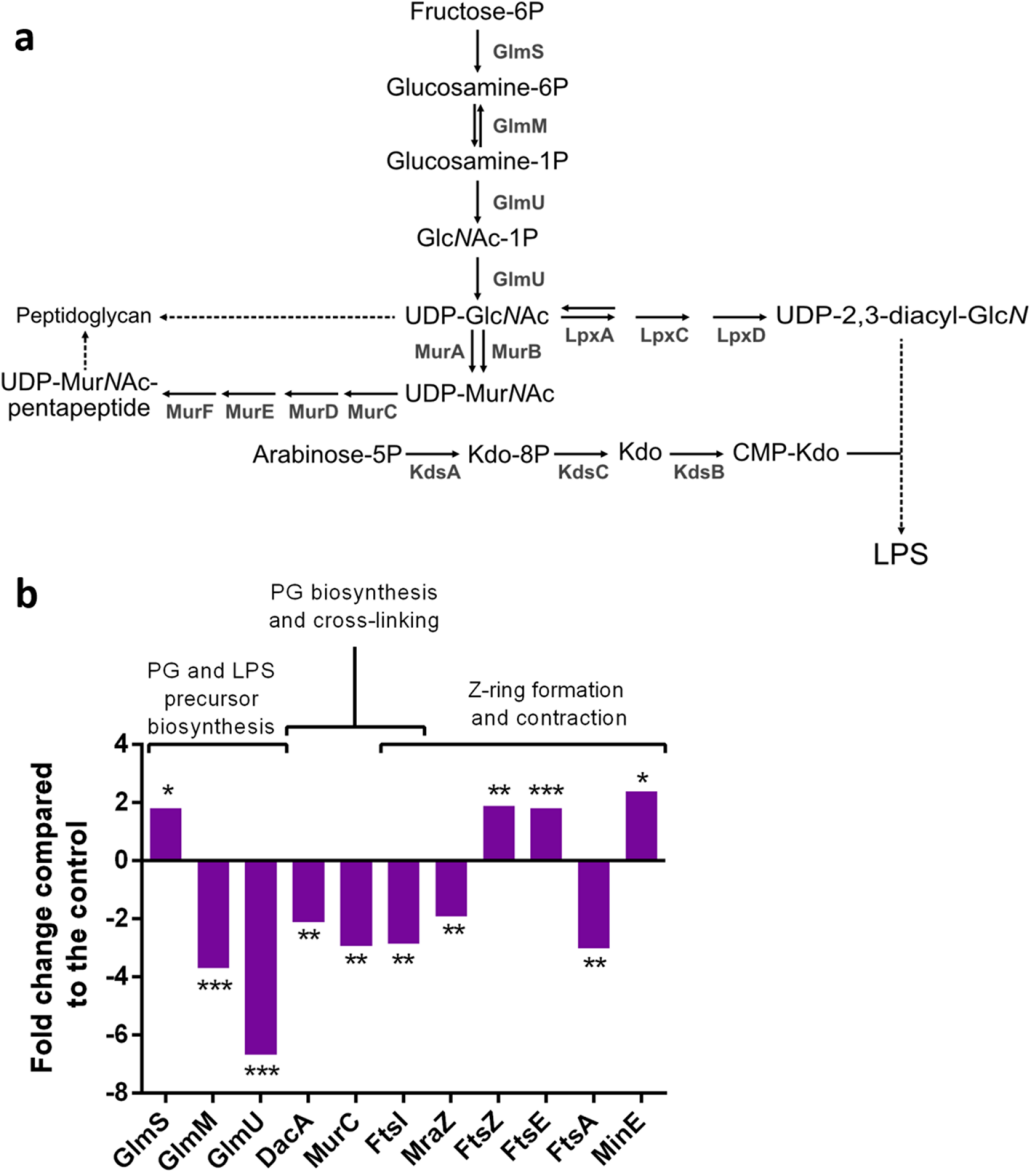
Modifications in the proteomic profile of MFAF76a relative to envelope component synthesis and z-ring formation after exposure to 45 ppm of NO_2_. (**a**) Biosynthesis pathways of peptidoglycan and lipopolysaccharides. (**b**) Changes in the abundance of proteins involved in the synthesis and metabolism of various components of the bacterial envelope, as well as in the cell cycle, after exposure to 45 ppm of NO_2_. The proteins were identified using the *P. fluorescens* A506 strain as reference (N = 4). * = *p* < 0.05; ** = *p* < 0.01; *** = *p* < 0.001.

To get further insights into the potential causes of the NO_2_-induced alterations on cell morphology of *P. fluorescens* MFAF76a, a global proteomic profiling analysis was performed comparing NO_2_ or synthetic air exposure of bacteria. The proteomic data of MFAF76a has revealed the modulation of the quantities of several proteins involved in the formation of the components of the bacterial envelope and Z-ring formation (Fig. 2b).

The proteomic study data revealed variations in the quantity of proteins involved in the synthesis of uridine diphosphate *N*-acetylglucosamine (UDP-Glc*N*Ac), the precursor of peptidoglycan and lipopolysaccharides (Fig. 2a). These proteins are GlmS, GlmM and GlmU. The results also revealed decreased amounts of FtsI, DacA and MurC, all involved in peptidoglycan synthesis and cross-linking after exposure to NO_2_.

The proteome study analysis also revealed important amount modifications variations of the proteins MraZ, FtsZ, FtsE, FtsA, FtsI and MinE playing a role in the formation of the septal ring^29^.

### NO_2_ exposure alters* P. fluorescens* MFAF76a membrane fluidity and integrity

**Figure 3:**
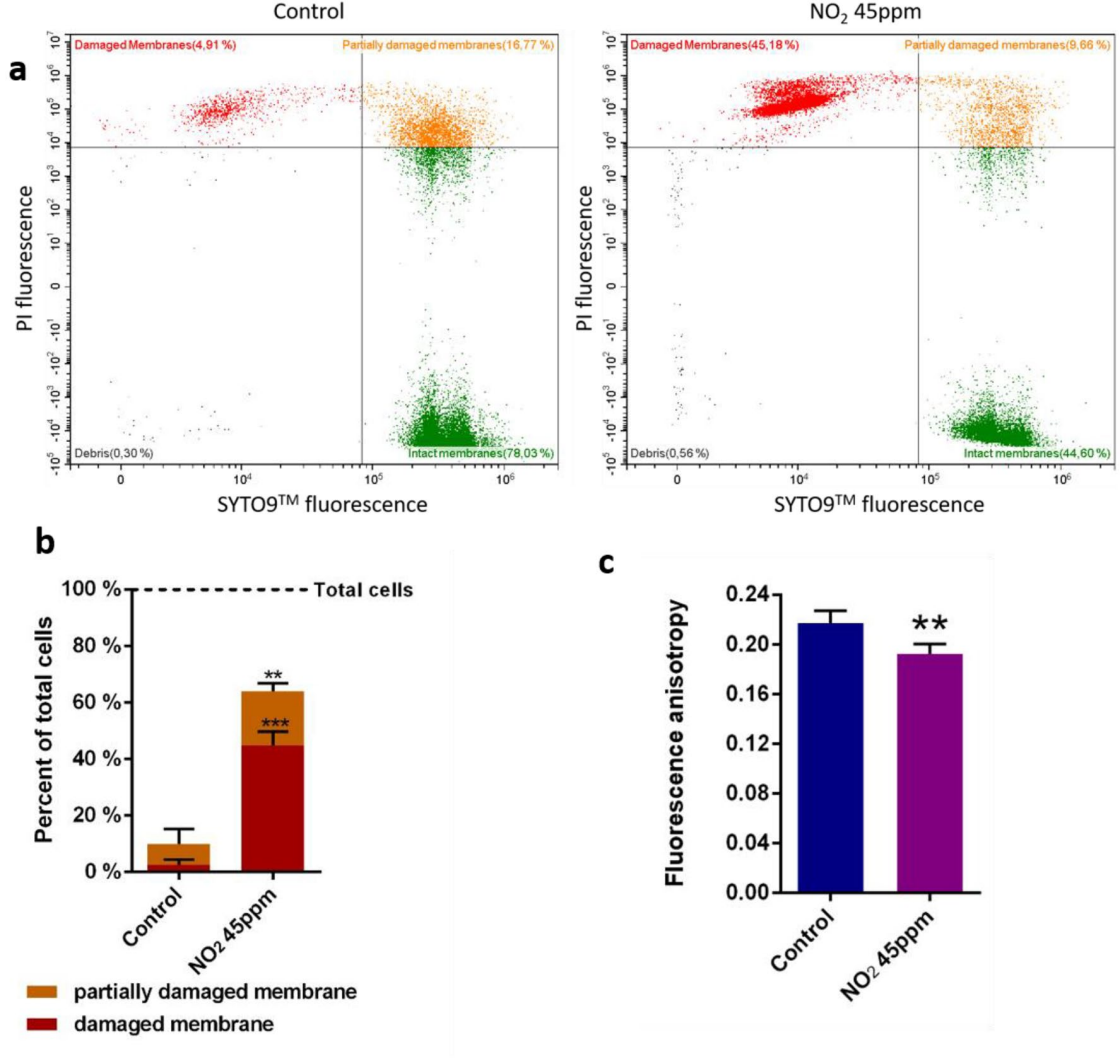
Membrane fluidity and integrity of *P. fluorescens* MFAF76a upon exposure to NO_2_. Bacterial cells were exposed to 45 ppm of NO_2_ (NO_2_ 45 ppm) or to synthetic air (Control) for 2 h. (**a**) Intensity of cell fluorescence in the PI and SYTO9 spectra of emission. (**b**) Membrane integrity assessed by live-dead flow cytometry assays using live-dead kit according to the manufacturer’s recommendations. (**c**) Membrane fluidity was determined by fluorescence anisotropy at 28 °C using DPH probe. Statistical significance was determined using t-test (N ≥ 4). ** = *p* < 0.01; *** = *p* < 0.001.

Aside from peptidoglycan, the other critical components in the structure of the cell envelope are the cell membranes. To clarify whether the effects of NO_2_ observed by electronic microscopy and flow cytometry are also mediated by membrane alterations, membrane integrity and membrane fluidity were assessed for bacteria exposed to NO_2_ or synthetic air. Membrane integrity assays were performed using the green-fluorescent membrane-permeant DNA probe SYTO9™ and the red-fluorescent membrane impermeant DNA probe propidium iodine (PI). Their fluorescence was measured by flow cytometry (Fig. 3b). Three populations of cells were distinguishable after labelling (Fig. 3a). Cells with intact membranes were only labeled by SYTO9 ™, while cells with a damaged membrane were labelled by both PI and SYTO9™. However, cells labelled by PI did not form a single population. Some cells formed a highly homogenous population displaying high red fluorescence and a green fluorescence in the lower ranges of SYTO9™ labelled cells (damaged membranes, Fig. 3a). This profile was similar to the control cells permeabilized by 50% ethanol, suggesting that their membranes completely lost their integrity. However, another, less homogenous, population of cells was fluorescent in the red and displayed a higher and more heterogeneous green fluorescence (partially damaged membranes, Fig. 3a). This profile was not found in the permeabilized cell control, suggesting that the membranes of these cells were only partially damaged. The PI fluorescence threshold used to discriminate between the partially damaged and the intact cells was put at the maximum PI fluorescence detected in non-labelled cells, sometimes resulting in the splitting of this population between partially damaged membranes and intact membranes. The SYTO9™ fluorescence threshold to discriminate between partially and totally damaged membranes was determined thanks to the cells permeabilized by 50% ethanol. Interestingly, the results showed a dramatic increase of the number of cells labelled by PI upon exposure to NO_2_. Indeed, 42.5% of the cells exposed to NO_2_ were damaged, as compared to 2.5% of the control cells (Fig. 3b). Meanwhile, 18.2% of the exposed bacteria and 7.1% of the control bacteria (Fig. 3b) displayed the profile associated with partially damaged membranes. Membrane fluidity of *P.* fluorescens MFAF76a was determined by measuring the fluorescence anisotropy of the 1,6-Diphenyl-1,3,5-hexatriene (DPH) fluorescent probe (Fig. 3c). DPH is hydrophobic and localizes at the core of the phospholipid bilayer, at the level of the fatty acid chains^30^. Excitation of the probe by polarized light quantifies the polarization of emitted fluorescence. The polarization of emitted fluorescence depends on the capacity of the probe to rotate upon itself. Therefore, the fluorescence polarization is proportional to the fluidity of the lipid bilayer in which the probe is inserted^31,32^. DPH behavior can also be affected by its ratio to lipids, but previous gas chromatography analysis did not show any difference in terms of quantity, composition and cis/trans ratio of fatty acid between the two conditions^33^. Our results showed that fluorescence anisotropy was significantly reduced to 0.192 after bacterial exposure to NO_2_ (45 ppm) as compared to 0.217 for control condition (synthetic air), indicating an increased membrane fluidity (Fig. 3c).

**Figure 4:**
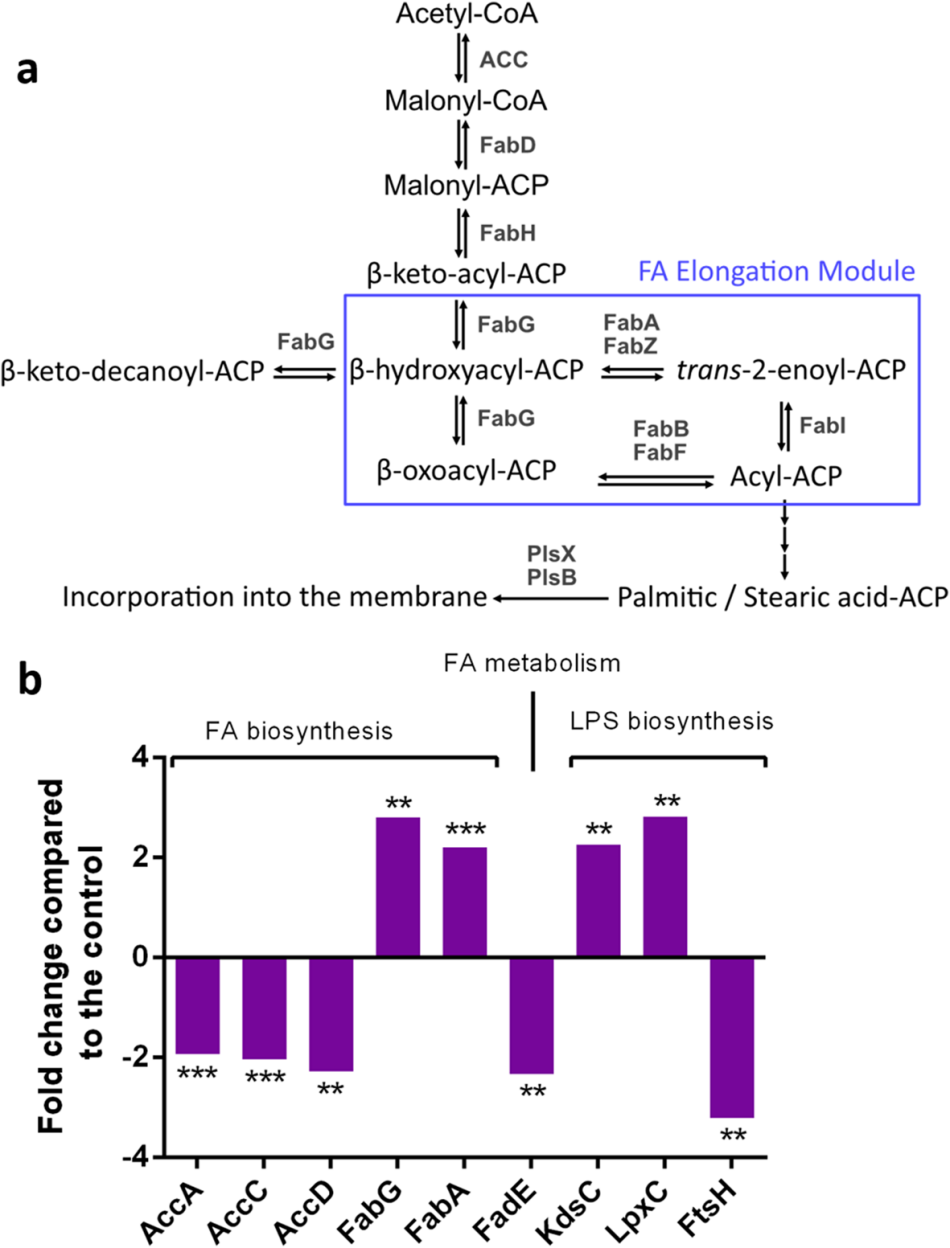
Modifications in the proteomic profile of MFAF76a relative to fatty acid and LPS biosynthesis and metabolism after exposure to 45 ppm of NO_2_. (**a**) Biosynthesis pathways of fatty acids in *Pseudomonas spp*. (**b**) Changes in the abundance of proteins involved in the synthesis and metabolism of fatty acids and lipopolysaccharide after exposure to 45 ppm of NO_2_. The proteins were identified using the *P. fluorescens* A506 strain as reference (N = 4). * = *p* < 0.05; ** = *p* < 0.01; *** = *p* < 0.001.

To clarify the consequences of the membrane alterations observed by cytometry and anisotropy, a proteomic profiling analysis of the proteins involved in the synthesis of membrane components was performed (Fig. 4). KdsC and LpxC, two proteins involved in the early steps of lipopolysaccharides biosynthesis were more detected after exposure to NO_2_. Furthermore, three proteins involved in the formation of the acetyl-CoA-carboxylase complex (ACC), responsible for the early step of fatty acid biosynthesis, were present in lower amounts in the condition exposed to NO_2_. These proteins are AccA, AccC and AccD. Furthermore, FabG and FabA, involved in latter steps of fatty acid biosynthesis were overproduced after NO_2_ exposure. Finally, FadE, involved in the first step of fatty acid metabolism through beta-oxidation was found in lower amounts compared to the control condition. However, proteins involved in phospholipid biosynthesis were not identified.

In a previous study, we showed slight modifications in the phospholipid composition of *P. fluorescens* MFAF76a membranes after exposure to NO_2_, including the disappearance of a minor unknown glycerophospholipid (UGP), but no lipid peroxidation seems to take place^33^. These previous results combined with the observed membrane alterations noted in Fig. 1 suggest that NO_2_ exposure could have an impact on the cell phospholipids. By searching the genome of MFAF76a strain, ten genes involved in the different steps of phospholipid biosynthesis from glycerol-3-phosphate (glycerol-3P) and acetyl-coenzyme A (acetyl-CoA) were found (*cdsA, clsA, clsB, clsC, pgpA, pgsA, pssA, plsC, psd* and *pcs*) (Fig. S1a). Since the proteins involved in phospholipid biosynthesis in *P. fluorescens* were not detected by the proteomic profiling analysis, the transcriptional regulation of the genes encoding for these proteins was assessed (Fig. S1b). RT-qPCR assays (Fig. S1b) showed an important transcription decrease of *clsB*, encoding the cardiolipin synthase B (ClsB). However, no significant expression levels difference was noted for the other studied genes upon NO_2_ exposure.

## Discussion

NO_2_ is an important air pollutant whose effects on the human lung microbiota are poorly understood. To study the effects of atmospheric NO_2_ on bacteria, the airborne *P. fluorescens* MFAF76a strain was exposed to either 45 ppm of gaseous NO_2_ or synthetic air for 2 h, through an exposition system described previously^21^.

In this study, electronic microscopy observations revealed that exposure to 45 ppm of NO_2_ affects the envelope cell morphology of MFAF76a strain (Fig. 1a). Indeed, while some control cells displayed a rough cell surface, the prevalence and intensity of these irregularities were much higher among cells exposed to NO_2_. The effect of NO_2_ on the bacterial envelope is reminiscent of the study of Deupree and Schoenfisch that showed the deterioration of *P. aeruginosa* membranes by NO, and outlined even potential holes formation^34^, and suggests that NO_2_ could alter membranes in a similar fashion. In this study, we tried to explain the emergence of the observed phenotype and to understand the effects of NO_2_ on the various cell components.

Flow cytometry showed that exposure to NO_2_ induces an increase in cell size (Fig. 1b,c). However, the cell size did not increase homogeneously, as the cells exposed to NO_2_ displayed more high sized events (Fig. 1b,c). This increase of the number of cells of high size could be the result of an alteration of the cell division process. This alteration is however temporary as the generation time of the bacteria published in Depayras et al. were not different after exposure to NO_2_^33^. The growth curve displayed a lag phase, which was at least partially explained by a significant diminution of cell viability.

To gain further insights into the observed cellular morphology modifications in MFAF76a strain, a proteome profiling approach was assessed in the absence or presence of NO_2_. Interestingly, the proteomic profiling data (Fig. 2b) revealed modifications of the amounts of several key proteins involved in the formation of the septal ring after exposure to NO_2_, supporting the results found by flow cytometry (Fig. 1b). Septal ring formation is a complex process. Briefly, FtsZ polymerizes into a z-ring at the midcell and recruits other proteins such as FtsA and ZipA that anchor the z-ring to the membrane. This complex then recruits other effector proteins that will help in the contraction of the ring and the synthesis of cell wall at the division site to prevent the rupture of the cell. Z-ring localization is regulated by the Min complex, which prevents the polymerization of FtsZ near the poles of the cell^35^. Among the proteins involved in the bacterial divisome formation, six were differentially expressed upon exposure to NO_2_. These proteins are FtsA, FtsE, FtsI, FtsZ, MraZ and MinE. The protein FtsZ can polymerize into a protofilament forming the z-ring, and allows the recruitment of other effector proteins. This polymerization is inhibited at one cell pole by the MinCD complex, which is then separated by MinE, causing MinCD to reform at the opposite pole, only to be separated by MinE again. This oscillation of MinCD and MinE restricts the polymerization of FtsZ to the center of the cell. FtsE is part of the transmembrane FtsEX complex, an ABC transporter-like protein required for cell division in low salt environment^36^. This complex is located to the septal ring through FtsE, while FtsX has multiple functions, including sequestration of FtsA to prevent its dimerization and recruitment of proteins involved in peptidoglycan hydrolysis^37^. The increase in FtsZ, MinE and FtsE quantities after NO_2_ exposure suggests the activation of regulatory pathways promoting cell division. However, another essential protein, FtsA, is under-expressed by threefold under nitrosative stress. FtsA is partly responsible for the attachment of the z-ring to the cell membrane as well as the recruitment of other downstream effector proteins necessary for cell constriction. Through its ability to bind FtsZ and the membrane, FtsA plays an important role in FtsZ dynamics. FtsA acts as a membrane anchor for FtsZ, but also tends to destabilize FtsZ polymers, and an overproduction of FtsA impairs cell division. Furthermore, FtsA is also able to self-polymerize which could inhibit its functions. This process could not be totally assumed since literature reports seemingly contradictory results on this subject^37–39^. Therefore, several hypotheses could explain the sharp decrease of the quantity of FtsA inside the cell after exposure to NO_2_. Lowering the production of FtsA could be a way for the cell to improve the septal ring stability by promoting FtsZ polymerization. FtsA could also be degraded after failing to attach to a membrane disorganized by NO_2_. Finally, FtsI abundance is also decreased by threefold. This protein is responsible for the synthesis of peptidoglycan at the septal ring location. This biosynthesis allows the contraction of the membrane without destroying the cell envelope. Normal localization of FtsI at the septum requires FtsA among other proteins. Since most proteins involved in the septal ring division are bound to the inner membrane, either directly or through their interaction with other proteins, perturbations of the membrane integrity could modulate their efficacy.

Furthermore, several proteins involved in the synthesis of peptidoglycan are differentially produced after exposure to NO_2_ (Fig. 2b). Thus, GlmS is overproduced while GlmM and GlmU are both underproduced in response to NO_2_ exposure. GlmS and GlmM reaction products are not specific to the peptidoglycan pathway, so GlmU decrease is a better indicator of the effect of NO_2_ regarding peptidoglycan synthesis. Peptidoglycan synthesis requires the presence of two precursors, uridine diphosphate *N*-acetylglucosamine (UDP-Glc*N*Ac) and uridine diphosphate *N*-acetylglucosamine (UDP-Mur-*N*Ac)^40^. Furthermore, MurC, involved in the addition of the pentapeptide moiety to UDP-Mur-*N*Ac is also underproduced. Finally, FtsI, responsible for peptidoglycan cross-linking at the site of the division septum, and DacA, which mediates peptidoglycan cross-linking are less abundant^41,42^. These results show that peptidoglycan synthesis is underproduced upon exposure to NO_2_ through the limitation of the synthesis of its precursor. Furthermore, a decrease in DacA abundance can alter bacterial morphology, by producing more irregular cells^43^. The decreased DacA quantity after exposure to NO_2_ could therefore contribute to the cell surface irregularities observed by electron microscopy.

The morphological alterations could also be linked to modifications of the membranes. To ascertain if the cell surface and elongated cells phenotypes are associated to membrane integrity alterations, membrane integrity measurements were evaluated in MFAF76a strain in the absence or presence of NO_2_ (Fig. 3a, b). The integrity data showed that membranes of 42.5% of the cells exposed to NO_2_ were completely permeant to PI, while being only weakly labelled by SYTO9™. Meanwhile, 18.2% of the cells exposed to NO_2_ showed strong labelling with both PI and SYTO9™ (Fig. 3b). This co-labelling indicates that the cells where less permeant to PI, suggesting their membranes had a higher level of integrity than the cells entirely permeant to PI. These values, compared with the respective 2.5% and 7.1% of the control cells, suggest that the cell morphology alterations observed by electron microscopy could be correlated to a decrease of the membrane integrity. These results prompted us to also explore the membrane fluidity which was quantified by fluorescence anisotropy using the DPH probe (Fig. 3c). Exposure to NO_2_ decreased significantly DPH fluorescence anisotropy in MFAF76a, indicating an increase of membrane disorganization. This increase could be the result of alterations to the membrane phospholipids, as well as damages to membrane proteins caused by NO_2_ and its potential derivatives. Together, these results confirm that the membranes of the MFAF76a strain were strongly altered upon NO_2_ exposure.

The proteomic profiling analysis of proteins involved in the synthesis of membrane components revealed that these pathways are also affected by NO_2_ (Fig. 4b). UDP-Glc*N*Ac, involved in an early step of PG synthesis, is also the precursor of lipid A, a part of lipopolysaccharide (LPS) when associated with a polysaccharide. The first stage of the LPS biosynthesis pathway is the conversion of UDP-Glc*N*Ac into Kdo_2_-lipid A by nine enzymes^44^. The protein LpxC, which is strongly, overproduced after exposure to NO_2_, catalyses the first committed step of this conversion. Synthesis of Kdo_2_-lipid A also requires the addition of two CMP-3-deoxy-D-manno-octulosonic acid (CMP-Kdo) residues^44^. KdsC is the second of the three enzymes of the subpathway synthetizing CMP-Kdo from D-ribulose-5-phosphate^45^. This enzyme is also overproduced in cells exposed to NO_2_. Taken together, these results suggest that NO_2_ had an effect on synthesis of the peptidoglycan and lipopolysaccharides precursor UDP-Glc*N*Ac.

Furthermore, the quantity of several proteins involved in fatty acid biosynthesis (FAB) was modulated in response to NO_2_ exposure (Fig. 4b). ACC is a four-subunit complex catalysing the first step of FAB in *Pseudomonas*^46^. Three of these subunits, AccA, AccC and AccD, were underproduced in the cells exposed to NO_2_, suggesting decreased FAB. Two proteins involved in the elongation of fatty acid, FabA and FabG, are overproduced after NO_2_ exposure (Fig. 4a). Taken together, these results indicate that NO_2_ exposure altered FAB pathway in MFAF76a strain. The overproduction of FabA could cause the increase of cis-unsaturated fatty acids, leading to an increase of membrane fluidity. However, the increase of membrane fluidity is unlikely to be caused by increase of cis-UFA production, since in a previous studies we showed that NO_2_ exposure did not induce any significant alteration of the degree of saturation nor in cis–trans isomerization ratio of fatty acids^33^. Moreover, FadE, which catalyzes the first step of fatty acid beta-oxidation, was underproduced after exposure to NO_2_^47^, indicating that the beta-oxidation of fatty acids is also impacted by NO_2_ and may be downregulated. Interestingly, despite the modification observed in membrane components biosynthesis, the bacterial colonies displayed no observable differences between the control and the cells exposed to NO_2_.

The proteomic profiling study showed that gaseous NO_2_ have an important effect on proteins synthesizing most of the envelope components. However, abundance variations on proteins involved in phospholipid biosynthesis was not detected with this approach. To assess whether bacteria modulate their phospholipid biosynthesis in response to NO_2_, the transcription of genes encoding for enzymes involved in this process were analyzed by RT-qPCR. No significant differences in expression levels of genes involved in phospholipid biosynthesis between control cells and NO_2_ exposed cells were found except for *clsB*, the expression of which was reduced by eightfold. *clsB* encodes for the cardiolipin synthase B protein, involved in the last step of the cardiolipin biosynthesis pathway, and is only active in stationary phase of growth^48^. Despite the presence of three cardiolipin synthase genes in the MFAF76a strain, no cardiolipin was characterized in the lipidomic profile of the MFAF76a strain^33^. However, it is possible that the absence of cardiolipin could be due to the methods used to extract and analyze the cardiolipin profile. Cardiolipin is known to have an important role in membrane structure, by increasing membrane fluidity or by producing a favorable environment for the correct functioning of membrane proteins^49^. Cardiolipin is also known to be localized preferentially at the poles of the cell. Therefore it plays a role in determining the localization of proteins involved in the septal ring formation such as the Min complex by favoring its biding to the membrane at the cell poles^50,51^. These data show that exposure to NO_2_ affected the cardiolipin biosynthesis pathway, which might lead to a reconfiguration of the bacterial envelope, in accordance with UGP loss upon NO_2_ exposure.

Overall, these results show that NO_2_ exposure of MFAF76a leads to an augmentation of the cell size, which may be linked to divisome formation impairment. Furthermore, NO_2_ induced envelope alterations and decrease membrane integrity. These alterations can be partially explained by the modification of numerous components of the cell envelope, including peptidoglycan, lipid A and phospholipids. Further experiments on other bacterial strains would permit the distinction between the general effect of NO_2_ on bacteria and the effects that may be specific to this strain.

## Supplementary Information

Supplementary Information.

## Data Availability

The datasets used and/or analyzed during the current study are available from the corresponding author on reasonable request.
