# Supplementary material for: Gaseous NO2 induces various envelope alterations in Pseudomonas fluorescens MFAF76a
Source: Sci Rep. 2022 May 20;12:8528. doi: 10.1038/s41598-022-11606-w (PMC9122911; doi:10.1038/s41598-022-11606-w)
Supplement: Supplementary file 1 — Supplementary Information. [file 41598_2022_11606_MOESM1_ESM.docx]

**Table S1.** Changes in the abundance of proteins involved in the synthesis and metabolism of various components of the bacterial envelope, as well as in the cell cycle, after exposure to 45 ppm of NO_2_. The proteins were identified using the *P. fluorescens* A506 strain as reference (N=4).

| **Biological Pathway** | **RefSeq**  **accession**  **number** | **Protein name** | **Product** | **Fold Change** | **ANOVA (p)** |
| --- | --- | --- | --- | --- | --- |
| Peptidoglycan and Lipopolysaccharide precursor biosynthesis | WP_014720585 | GlmS | glutamine-fructose-6-phosphate transaminase, isomerizing | +1.80 | 1.29E-02 |
|  | WP_003194195 | GlmM | phosphoglucosamine mutase | -3.70 | 6.02E-04 |
|  | WP_014720587 | GlmU | UDP-N-acetylglucosaminediphosphorylase | -6.67 | 7.27E-04 |
| Peptidoglycan biosynthesis and cross-linking | WP_003188665 | FtsI | peptidoglycansynthetase FtsI | -2.86 | 5.35E-03 |
|  | WP_014720083 | DacA | D-alanyl-D-alanine carboxypeptidase | -2.12 | 2.11E-03 |
|  | WP_003188677 | MurC | UDP-N-acetylmuramate--L-alanine ligase | -2.94 | 5.46E-03 |
| Z-ring formation and contraction | WP_003171868 | MraZ | MraZ protein | +1.92 | 3.28E-03 |
|  | WP_014716977 | FtsZ | cell division protein FtsZ | +1.90 | 3.34E-03 |
|  | WP_003234627 | FtsE | cell division ATP-binding protein FtsE | +1.82 | 1.82E-04 |
|  | WP_014716976 | FtsA | cell division protein FtsA | -3.03 | 1.34E-03 |
|  | WP_003188665 | FtsI | peptidoglycansynthetase FtsI | -2.86 | 5.35E-03 |
|  | WP_003175252 | MinE | cell division topological specificity factor MinE | +2.39 | 1.15E-02 |

**Table S2.** Changes in the abundance of proteins involved in the synthesis and metabolism of fatty acids and lipopolysaccharide. The proteins were identified using the *P. fluorescens* A506 strain as reference (N=4).

| **Biological Pathway** | **RefSeq**  **accession**  **number** | **Protein name** | **Product** | **Fold Change** | **Anova (p)** |
| --- | --- | --- | --- | --- | --- |
| Fatty acid biosynthesis | WP_003189182 | AccA | acetyl-CoA carboxylase, carboxyltransferase subunit alpha | -1.92 | 8.34E-05 |
|  | WP_003195732 | AccC | acetyl-CoA carboxylase, biotin carboxylase subunit | -2.04 | 6.90E-04 |
|  | WP_014719154 | AccD | acetyl-CoA carboxylase, carboxyltransferase subunit beta | -2.27 | 4.33E-03 |
|  | WP_005790544 | FabG | 3-oxoacyl-ACP reductase | 2.81 | 4.91E-03 |
|  | WP_003190035 | FabA | 3-hydroxydecanoyl-ACP dehydratase | 2.21 | 3.66E-04 |
| Fatty acid metabolism | WP_014717779 | FadE | acyl-coenzyme A dehydrogenase | -2.33 | 2.67E-03 |
| Lipopolysaccharide biosynthesis | WP_014716938 | KdsC | 3-deoxy-D-manno-octulosonate 8-phosphate phosphatase | 2.26 | 8.05E-03 |
|  | WP_003171886 | LpxC | UDP-3-O-[3-hydroxymyristoyl] N-acetylglucosamine deacetylase | 2.82 | 4.41E-03 |
|  | WP_003189182 | FtsH | ATP-dependent metalloprotease FtsH | 3.21 | 1.66E-03 |

| 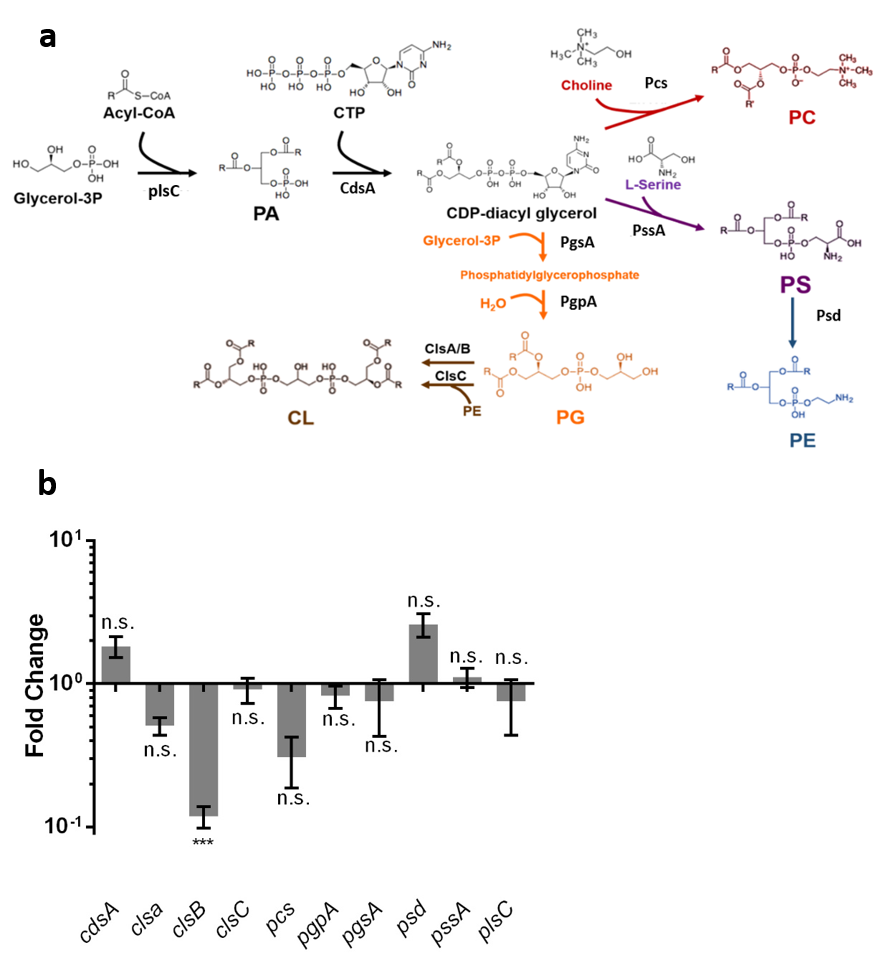 |
| --- |
|  |
|  |

**Fig. S1.** Expression of genes coding for phospholipid biosynthesis pathway proteins. (a) Phospholipid biosynthesis pathways in *P. fluorescens*. (b) Expression of genes involved in phospholipid synthesis after exposure to gaseous NO_2_ (45 ppm). The effect of NO_2_ exposure on the expression of the ten genes coding for proteins involved in phospholipids biosynthesis was assessed by RT-qPCR. The representation is based on the relative gene expression of MFAF76a strain exposed to 45 ppm of NO_2_ compared to the air control condition (synthetic air). Statistical significance was determined using unpaired t-test (N=5). n.s.=p>0.05; ***=p <0.001.
